# Relationship between Diet and Mental Health in a Young Adult Appalachian College Population

**DOI:** 10.3390/nu10080957

**Published:** 2018-07-25

**Authors:** Rachel A. Wattick, Rebecca L. Hagedorn, Melissa D. Olfert

**Affiliations:** 1 Division of Animal and Nutritional Sciences, Davis College of Agriculture, Natural Resources and Design, West Virginia University, 4100 Agricultural Sciences Building, P.O. Box 6108, Morgantown, WV 26505-6108, USA; rawattick@mix.wvu.edu (R.A.W.); rlhagedorn@mix.wvu.edu (R.L.H.); 2 Human Nutrition and Foods, Division of Animal and Nutritional Sciences, Davis College of Agriculture, Natural Resources and Design, West Virginia University, G25 Agricultural Sciences Building, 333 Evansdale, Morgantown, WV 26505-6108, USA

**Keywords:** mental health, young adult, diet quality, food insecurity, college, student

## Abstract

Young adults in Appalachia may face poor nutritional status due to low access to healthy food and high mental health symptoms attributed to high stress and the college environment. A cross-sectional design was used to investigate the relationship between diet intake and mental health status of this population via surveys. Participant responses (*n* = 1956) showed students’ mean number of depressed days over the past 30 days was 9.67 ± 8.80, and of anxious days, 14.1 ± 10.03. The mean fruit and vegetable intake was 1.80 ± 1.27 times per day and the mean added sugars intake was 1.79 ± 1.26 times per day. 36.7% of students were found to be food insecure. One-way ANOVA and Chi-Squared analyses were used to determine relationship between variables. Significant variables were placed into a full logistic regression model. Food insecurity and fruit and vegetable intake remained significant predictors of depression in males (odds ratio (OR) = 2.33 95% CI 1.47–3.71 and OR = 68 95% CI 50–89, respectively) and in females food insecurity remained a significant predictor of depression (OR = 2.26 95% CI 1.67–3.07). Food insecurity and added sugars intake were significant predictor of anxiety in males (OR = 2.33 95% CI 1.47–3.71 and OR = 1.09 95% CI 0.91–1.3, respectively) and for anxiety in females, added sugars intake and food insecurity were significant predictors (OR = 1.18 95% CI 1.05–1.32 and OR = 1.65 95% CI 1.27–2.16, respectively). Improving college student’s diet intake through increased access to healthy foods could improve the mental health and well-being of students.

## 1. Introduction

Mental health disorders affect 22.1% of the U.S. young adult population (ages 18–25), which is the highest prevalence of any age group [1]. Two common disorders in young adults are major depression and anxiety, occurring at 10.9% and 22.3%, respectively [1]. These disorders are often attributed to a serotonin deficiency and are treated with medications such as Selective Serotonin Reuptake Inhibitors (SSRIs). Medications are not always favored by patients, who report a three-fold preference for non-pharmacological forms of treatment [2]. Historically, nutrition has been overlooked as a contributor to poor mental health, but there is an increasing focus on this relationship, largely due to the central nervous system’s need for key nutrients to maintain optimal function. The nutritional status of an individual is influenced by a variety of factors, including: Life stage, environment, food access, and socioeconomic status [3]. In turn, each of these factors can influence mental health [4].

The majority of research investigating the relationship between nutrition and mental health has been focused on older adults. There are some studies that have focused on food insecurity across the lifespan and mental health [5,6,7,8,9,10], and specific nutrients’ effects on mental health [11,12,13,14,15,16,17,18,19], while some have focused on overall dietary quality and mental health [20,21,22]. It may be more beneficial to examine overall dietary intake’s relationship to mental health, as this is more representative of human lifestyle behaviors. College-attending young adults face a variety of risk factors for poor mental health, including high stress and high rates of food insecurity [23]. Food insecurity is defined as the lack of consistent access to a sufficient quantity of healthy affordable foods; on college campuses rates of food insecurity among students have been reported to be as high as 59% [23]. Importantly, food insecurity has been shown to be associated with poor mental health [5,6,7,8,9,10]. Food insecurity rates can vary by region and are particularly high in the Appalachian area, with rates as high as 20.8%, much higher than the US average of 12.3% [24]. The Appalachian region spreads over 13 states in the southern and northeastern regions of the US, and has been traditionally plagued by health disparities due to its rural landscape and difficult transportation from being situated in the Appalachian mountains, making access to healthcare difficult [25]. The dietary intake of depressed or anxious individuals has been minimally studied but have shown to vary regionally [4]. Because of these differences in dietary habits, the prevalence or severity of mental health symptoms may vary regionally as well. Overall, there is a limited amount of data available on the health of Appalachian individuals, who are at high risk of poor health due to low socioeconomic status [25], low food access [24]. and low access to health services such as to counseling with registered dietitians [25]. These and additional poor health risks, such as stress, are present on college campuses. Due to the many risk factors for poor mental health and nutritional shortcomings faced by young adults on an Appalachian college campus, the prevalence of anxiety and depression and its relationship to diet intake needs to be studied.

There is a strong relationship between nutrition and mental health in adults [26,27,28,29,30] and has been found to differ between depressed and anxious individuals [26,27,28]. Further, diet intake has been found to differ between males and females with mental health disorders. For example, previous work has found that depressed or anxious females consume greater quantities of unhealthy foods than depressed or anxious males [26,27,28]. However, no studies have yet identified this relationship in a US young adult population in an Appalachian college setting.

Therefore, the aims of this study was to determine the frequency of depression and anxiety symptoms in an Appalachian college-attending young adult population and the relationship between these symptoms and dietary intake. In addition, the role of food insecurity in relationship to these factors was examined. It is hypothesized that (1) Individuals with poor mental health, classified as those with high frequency of depression and anxiety symptoms would show different diet intake characterized by lower fruit and vegetable intake and higher sugar intake, (2) individuals categorized as food insecure would show higher days of poor mental health and (3) there would be differences in diet intake between genders, and between depressed and anxious individuals.

## 2. Materials and Methods

### 2.1. Design

This cross-sectional study investigated a convenience sample of young adults attending a large, Appalachian university in fall 2017. To be eligible, participants had to be currently enrolled at the university and at least 18 years of age.

### 2.2. Ethics Approval and Consent to Participate

This study was approved by West Virginia University’s Institutional Review Board (#170350219) in accordance with the Declaration of Helskini. All subjects gave their informed consent for inclusion before participating.

### 2.3. Participants and Procedures

Undergraduate and graduate students attending a large, Appalachian university in the fall 2017 semester were recruited. Using the university listserv, students were emailed an online survey link which directed them to Qualtrics, an anonymous online survey platform. Participants read the informed consent, and if accepted, completed the survey. Students who denied consent were exited from the page. Students were incentivized to take the survey via the chance to win one of four $100 American Express gift cards. Contact information remained separate from the results of the survey to maintain confidentiality.

### 2.4. Survey Design

The 116-item survey was developed by researchers from a multistate, collaborative university research group. The survey asked participants to self-report their sex, height, and weight. Students were screened for depression and anxiety symptoms using items from the Center for Disease Control and Prevention’s Healthy Days Measure [31], for food insecurity using the US Adult Food Security Survey Module [32], and for dietary intake using the Dietary Screener Questionnaire (DSQ) [33]. The Healthy Days Measure is a validated tool used to assess the prevalence and symptoms of mental distress [31]. The US Adult Food Security Survey Module is a validated tool to screen for food insecurity in adults and asks respondents to choose whether statements such as “In the past 30 days, the food I bought just didn’t last, and I didn’t have money to get more” or “I couldn’t afford to eat balanced meals” were often true, sometimes true, or never true. The DSQ groups responses into a dietary score for different food groups. Food groups examined for this study were fruit and vegetable intake and added sugars, due to fruit and vegetable consumption being considered representative of overall diet quality [29], and sugar consumption being shown to possibly be detrimental to mental health [34,35]. For the fruit and vegetable domain, items included: Fruit, fruit juice, salad, fried potatoes, other potatoes, dried beans, other vegetables, tomato sauce, salsa, and pizza. For the added sugars domain, items included: Soda, fruit drinks, cookies, cakes, pie, donuts, ice cream, sugar/honey in coffee/tea, candy, and cereal. Scores indicate frequency of daily consumption.

### 2.5. Statistical Analysis

Descriptive statistics were computed for all demographic and health questions. Participants were placed into depressed, not depressed, anxious, and not anxious categories using cutoffs of ≥14 days of feelings of depressed or anxious being designated as presence of depression or anxiety, as this cutoff is recommended by the CDC for their Health Days Measure [31]. For DSQ scoring, daily frequency of fruit and vegetable intake and added sugars intake were scored according to the DSQ scoring instructions. Upon review of the foods categorized as fruits and vegetables in this tool, fried potatoes, tomato sauce, salsa, and pizza were removed from the score, as these should not be counted towards fruit and vegetable intake goals, contributing mainly energy [36]. Food security status was scored using the USDA’s scoring system for the 10 questions. Scores with zero affirmative answers indicate high food security, scores of 1–2 marginal food security, 3–5 low food security, and 6–10 very low food security. Prevalence of food insecurity was determined by designating those in the high and marginal categories as food secure, and those in the low and very low categories as food insecure.

One-way ANOVA analysis was used to determine bivariate associations of depression and anxiety and BMI. Pearson Chi Squared analysis was used to determine associations of students with depression or anxiety and sex, housing, and food security status. All significant variables were placed into a full logistic regression model to determine the strongest predictor of poor mental health. Separate models were created for depression and anxiety. Models were stratified by sex due to the differences seen between the diet intake of males and females with poor mental health.

## 3. Results

The survey was completed by 1956 students. Respondents were predominantly aged 19–21 (59.4%) years old, female (67.5%), and from Appalachia (57.1%), and lived off campus (80.9%). The mean BMI of this sample was 25.2 ± 0.6 k/m^2^. Over a third (36.7%) of participants were found to be food insecure. The mean fruit and vegetable intake was 1.80 ± 1.3 times per day and the mean added sugars intake was 1.79 ± 1.3 times per day. The mean number of days feeling depressed was 9.67 ± 8.80, and the mean number of days feeling anxious was 14.1 ± 10.03.

There were associations between the presence of depression and sex (*p* = 0.0004), food security status (*p* ≤ 0.001), and added sugars intake (*p* = 0.228).

There were associations between presence of anxiety and sex (*p* ≤ 0.0001), food security status (*p* ≤ 0.0001), and added sugars intake (*p* = 0.005). Table 1 and Table 2 show sample characteristics by anxiety and depression status.

Results from full logistic regression models are presented in Table 3 and Table 4.

## 4. Discussion

To our knowledge, this is the first study to investigate the relationship between mental health, diet quality, and food insecurity in a young adult population attending an Appalachian university while examining differences between sexes. The mean number of days feeling depressed over the past 30 days was 9.67, and of feeling anxious, 14.1. These findings show that college students in the Appalachian environment face mental health problems for a third to half of the month, which may be hindering their academic progress and physical health. It was hypothesized that individuals with depression or anxiety would show lower fruit and vegetable intake and higher added sugar intake. This was true for fruit and vegetable intake in males with depression, and for added sugar intake in males and females with depression. It was also hypothesized that food insecure individuals would show higher prevalence of depression and anxiety. This was found to be true for depression and anxiety in both males and females. Finally, it was hypothesized that there would be a difference in dietary intake of those with depression and anxiety, and between sexes. This was found to be true, as depressed males showed lower fruit and vegetable intake, while depressed females did not, and anxious males and females showed higher added sugar intake.

When looking at determinants of mental health symptoms, there were differences between sexes. For males, the significant predictors of depression were fruit and vegetable intake and food insecurity, and for anxiety, the significant predictors were added sugars intake and food insecurity. For females, the significant predictor of depression was food insecurity, and for anxiety, the significant predictors were added sugars intake and food insecurity. These findings are consistent with previous studies showing differences in dietary patterns of males and females with mental health disorders [26,27,28]. These are also consistent with studies showing links between mental health and both dietary intake [26,27,28,29,30] and food insecurity [5,6,7,8,9,10], with lower intakes of healthy foods and higher rates of food insecurity showing an increase in prevalence of mental health symptoms.

Young adults face many factors for poor nutritional status and mental health. There are high rates of food insecurity and overall low access to healthy foods, high stress, and pressure to adapt and succeed in the college environment. These factors are increased when looking at college students on an Appalachian campus, a region plagued by poor health, low socioeconomic status, and poor access to health services [25]. The present study found that individuals on an Appalachian college campus face food insecurity at 36.7%, much higher than the national average. This is consistent with previous studies showing rates of food insecurity on college campuses to be much higher than the national average [23,37]. This is of concern for college campuses, as food insecurity has been linked to poorer physical and mental health [5,6,7,8,9,10]. These studies have hypothesized that food insecurity may be adding additional stress, contributing to worse mental health, and that food insecurity leads to lower dietary quality, providing the brain with less key nutrients for optimal function. The causes of this relationship need to be further determined, however the current body of knowledge has implications for current action by policymakers. Dietary interventions have shown that providing access to healthy foods can improve mental health symptoms, and thus interventions for this population should aim to provide healthy foods required for improved mental health [38,39,40]. In a study that examined a fruit and vegetable intervention on young adults, only those who were provided with a supply of fresh fruits and vegetables showed a significant increase in fruit and vegetable consumption and overall well-being [38]. Another study that provided tools and foods to depressed patients to follow a Mediterranean diet showed improvements in depression and anxiety symptoms and high adherence to the diet, even sustained at 6 months [39]. This highlights the importance of providing access to the healthy foods to enable individuals to adhere to a healthy diet. Thus, alleviating food insecurity on college campuses should be a priority in order to improve the well-being of students. This study adds to the body of work showing health disparities faced by Appalachia and the factors contributing to the mental health status of college students and provides a potential solution for improving their mental health.

There are a few limitations to the present study. First, we used self-reported mental health days rather than asking respondents if they have received a mental health diagnosis by a professional, because not all individuals with mental health disorders have received a medical diagnosis. The intention was to better capture data from those with symptoms of a mental health disorder. In addition, the DSQ includes items in the fruit and vegetable domain that are not healthy sources of fruits and vegetables, including fried potatoes and salsas, and thus were removed from the score in this study. This study focused on fruits and vegetables because they are considered to be indicative of overall diet quality [30], and on added sugars because of their evidence of being detrimental to mental health [32,33]. However, this study did not examine meat and saturated fats, which may be detrimental for mental health as well. Another limitation of the DSQ is that it accounted for frequency of consumption, or times per day, and not volume of consumption. Because of this, respondents’ consumption could not be compared to the Dietary Guidelines or national averages. Food insecurity was determined using a self-reported tool and is thus a perception, which may not reflect true food insecurity, and may not take into account other factors that cause a poor diet. The data used in this study was collected from one university, and thus may not be applied to every college environment in Appalachia. Student major was not accounted for, which could contribute to the stress of students as well as their health knowledge, potentially altering their behavior. Future work will include the use of a validated diagnostic tool, such as the Patient Health Questionnaire, and Beck Anxiety Inventory. In addition, the diet quality will be better screened for using a tool that encompasses a variety of foods with quantities consumed measured. Finally, data will be collected from multiple Appalachian college campuses.

To conclude, this study gathered data on a population that is understudied, despite their high risk for poor health due to low healthy food access, low socioeconomic status, and a high stress college environment. This study added data on the frequency and contributors to mental health symptoms in young adults on an Appalachian college campus. These individuals are at an important life-stage, where optimal physical and mental function is required to succeed. Identifying areas of need for this population is important to inform interventions. The results of this study are important to show that providing access to affordable healthy foods can improve the rates of mental health disorder symptoms in this environment. Access to healthy foods and dietary quality are associated with mental health, and these factors are modifiable. Improving these factors can improve the mental health of students, and thus may enhance their academic success.

## Figures and Tables

**Table 1:** Characteristics of respondents and correlations with depression status.

Variable	Depression		No Depression		*p*-Value
	*N*	%	*N*	%	
Total Population					
	329	30.3	756	69.7	
Sex					
Male	67	22.4	232	77.6	0.0004 *
Female	261	33.5	518	66.5	
Housing					
On-Campus	73	35.6	132	64.4	0.0725
Off-Campus	254	29.2	616	70.8	
Food Security Status					
Food Secure	146	23.1	486	76.9	<0.0001 *
Food Insecure	183	40.4	270	59.6	
	Mean	SD	Mean	SD	
BMI (kg/m^2^)	25.3	5.5	25.9	6.1	0.1970
Fruit/Vegetable Intake (times/day)	2.5	0.1	2.6	0.1	0.2694
Added Sugars (times/day)	1.9	0.1	1.7	0.0	0.0288 *

Demographic data presented in frequency and percentages. * *p* < 0.05.

**Table 2:** Characteristics of respondents and correlations with anxiety status.

Variable	Anxiety		No Anxiety		*p*-Value
	*N*	%	*N*	%	
Total Population					
	626	48.6	662	51.4	
Sex					
Male	115	33.5	228	66.5	<0.0001 *
Female	507	54.3	426	45.7	
Housing					
On-Campus	118	49.8	119	50.2	0.7415
Off-Campus	504	48.6	533	51.4	
Food Security Status					
Food Secure	334	42.9	445	57.1	<0.0001 *
Food Insecure	292	57.4	217	42.6	
	Mean	SD	Mean	SD	
BMI (kg/m^2^)	24.9	5.0	25.6	6.0	0.0568
Fruit/Vegetable Intake (times/day)	2.6	0.1	2.6	0.1	0.5842
Added Sugars (times/day)	1.9	0.0	1.7	0.0	0.0050 *

Demographic data presented in frequency and percentages. * *p* < 0.05.

**Table 3:** Logistic Regression model predicting depression status in male and female students.

	Male		Female	
Variable	Odds Ratio	95% CI	Odds Ratio	95% CI
Added Sugar Intake	1.14	0.93–1.40	1.09	0.95–1.24
FV Intake	0.68	0.50–0.89	0.94	0.83–1.06
Food Secure	0.50	0.28–0.88	0.44	0.33–0.60
Food Insecure	1.99	1.13–3.53	2.26	1.67–3.07

**Table 4:** Logistic regression model predicting anxiety status in male and female students.

	Male		Female	
Variable	Odds Ratio	95% CI	Odds Ratio	95% CI
Added Sugar Intake	1.09	0.91–1.30	2.08	0.43–0.84
Food Secure	0.43	0.27–0.68	0.43	0.27–0.68
Food Insecure	2.33	1.47–3.71	2.33	1.47–3.71

## References

[1] (2016). Mental Illness. https://www.nimh.nih.gov/health/statistics/mental-illness.shtml.

[2] Elliott J.O. (2016). Psychosocial interventions for mental and substance use disorders: A framework for establishing evidence-based standards, by institute of medicine (IOM). J. Soc. Work Pract. Addict..

[3] Darmon N., Drewnowski A. (2008). Does social class predict diet quality?. Am. J. Clin. Nutr..

[4] Stefanska E., Wendołowicz A., Cwalina U., Kowzan U., Konarzewska B., Szulc A., Ostrowska L. (2017). Assessment of dietary habits and nutritional status of depressive patients, depending on place of residence. Ann. Agric. Environ. Med..

[5] Martin M.S., Maddocks E., Chen Y., Gilman S.E., Colman I. (2016). Food insecurity and mental illness: Disproportionate impacts in the context of perceived stress and social isolation. Public Health.

[6] Pryor L., Lioret S., van der Waerden J., Fombonne É., Falissard B., Melchior M. (2016). Food insecurity and mental health problems among a community sample of young adults. Soc. Psychiatry Psychiatri. Epidemiol..

[7] Tarasuk V., Mitchell A., McLaren L., McIntyre L. (2013). Chronic physical and mental health conditions among adults may increase vulnerability to household food insecurity. J. Nutr..

[8] McLaughlin K.A., Green J.G., Alegría M., Costello E.J., Gruber M.J., Sampson N.A., Kessler R.C. (2012). Food insecurity and mental disorders in a national sample of US adolescents. J. Am. Acad. Child Adolesc. Psychiatry.

[9] Weaver L.J., Hadley C. (2009). Moving beyond hunger and nutrition: A systematic review of the evidence linking food insecurity and mental health in developing countries. Ecol. Food Nutr..

[10] Weinreb L., Wehler C., Perloff J., Scott R., Hosmer D., Sagor L., Gundersen C. (2002). Hunger: Its impact on children’s health and mental health. Pediatrics.

[11] Agostoni C., Nobile M., Ciappolino V., Delvecchio G., Tesei A., Turolo S., Crippa A., Mazzocchi A., Altamura C.A., Brambilla P. (2017). The Role of Omega-3 Fatty Acids in Developmental Psychopathology: A Systematic Review on Early Psychosis, Autism, and ADHD. Int. J. Mol. Sci..

[12] Berger M.E., Smesny S., Kim S.W., Davey C.G., Rice S., Sarnyai Z., Schlogelhofer M., Schafer M.R., Berk M., McGorry P.D. (2017). Omega-6 to omega-3 polyunsaturated fatty acid ratio and subsequent mood disorders in young people with at-risk mental states: A 7-year longitudinal study. Transl. Psychiatry.

[13] Gross G., Galvano F., Marventano S., Malaguarnera M., Bucolo C., Drago F., Caraci F. (2014). Omega-3 fatty acids and depression: Scientific evidence and biological mechanisms. Oxid. Med. Cell. Longev..

[14] Grosso G., Pajak A., Marventano S., Castellano S., Galvano F., Bucolo C., Drago F., Caraci F. (2014). Role of Omega-3 Fatty Acids in the Treatment of Depressive Disorders: A Comprehensive Meta-Analysis of Randomized Clinical Trials. PLoS ONE.

[15] Hallahan B., Ryan T., Hibbeln J.R., Murray I.T., Glynn S., Ramsden C.E., SanGiovanni J.P., Davis J.M. (2016). Efficacy of omega-3 highly unsaturated fatty acids in the treatment of depression. Br. J. Psychiatry.

[16] Jiao J., Li Q., Chu J., Zeng W., Yang M., Zhu S. (2014). Effect of n-3 PUFA supplementation on cognitive function throughout the life span from infancy to old age: A systematic review and meta-analysis of randomized controlled trials. Am. J. Clin. Nutr..

[17] Pusceddu M., Kelly P., Stanton C., Cryan J., Dinan T. (2016). N-3 Polyunsaturated Fatty Acids through the Lifespan: Implication for Psychopathology. Int. J. Neuropsychopharmacol..

[18] Rathod R., Kale A., Joshi S. (2016). Novel insights into the effect of vitamin B 12 and omega-3 fatty acids on brain function. J. Biomed. Sci..

[19] Skotheim S., Handeland K., Kjellevold M. (2017). The effect of school meals with fatty fish on adolescents’ self-reported symptoms for mental health: FINS-TEENS—A randomized controlled intervention trial. Food Nutr. Res..

[20] Carson N., Blake C., Saunders R. (2015). Perceptions and Dietary Intake of Self-Described Healthy and Unhealthy Eaters with Severe Mental Illness. Community Ment. Health J..

[21] Wilsher S.H. (2013). The impact of emotional health on fruit and vegetable consumption in young men: A qualitative study. Proc. Nutr. Soc..

[22] Deliens T., Clarys P., De Bourdeaudhuij I., Deforche B. (2014). Determinants of eating behaviour in university students: A qualitative study using focus group discussions. BMC Public Health.

[23] Cady C.L. (2014). Food insecurity as a student issue. J. Coll. Character.

[24] (2018). USDA ERS—Key Statistics & Graphics. https://www.ers.usda.gov/topics/food-nutrition-assistance/food-security-in-the-us/key-statistics-graphics.aspx.

[25] Behringer B., Friedell G.H. (2006). Appalachia: Where Place Matters in Health. Prev. Chronic Dis..

[26] Ohmori Y., Ito H., Morita A., Deura K., Miyachi M. (2017). Associations between depression and unhealthy behaviours related to metabolic syndrome: A cross sectional study. Asia Pac. J. Clin. Nutr..

[27] Stefanska E., Wendolowicz A., Cwalina U., Kowzan U., Konarzewska B., Szulc A., Ostrowska L. (2014). Assessment of dietary habits of patients with recurrent depressive disorders. Arch. Psychiatry Psychother..

[28] Meegan A.P., Perry I.J., Phillips C.M. (2017). The Association between Dietary Quality and Dietary Guideline Adherence with Mental Health Outcomes in Adults: A Cross-Sectional Analysis. Nutrients.

[29] Nguyen B., Ding D., Mihrshahi S. (2017). Fruit and vegetable consumption and psychological distress: Cross-sectional and longitudinal analyses based on a large Australian sample. BMJ Open.

[30] Jacka F.N. (2017). Nutritional Psychiatry: Where to Next?. EBioMedicine.

[31] Centers for Disease Control and Prevention Health-Related Quality of Life (HRQOL). https://www.cdc.gov/hrqol/concept.htm.

[32] Survey Tools. https://www.ers.usda.gov/topics/food-nutrition-assistance/food-security-in-the-us/survey-tools/#adult.

[33] Dietary Screener Questionnaire (DSQ) in the NHANES 2009-10: Dietary Factors, Food Items Asked, and Testing Status for DSQ. Long Isl. Breast Cancer Study Proj. (Past Initiat.).

[34] Molteni R., Barnard R.J., Ying Z., Roberts C.K., Gómez-Pinilla F. (2002). A high-fat, refined sugar diet reduces hippocampal brain-derived neurotrophic factor, neuronal plasticity, and learning. Neuroscience.

[35] Lim S.Y., Kim E.J., Kim A., Lee H.J., Choi H.J., Yang S.J. (2016). Nutritional Factors Affecting Mental Health. Clin. Nutr. Res..

[36] Cullen K.W., Baranowski T., Baranowski J., Hebert D., Moor C.D. (1999). Behavioral or Epidemiologic Coding of Fruit and Vegetable Consumption from 24-Hour Dietary Recalls. J. Acad. Nutr. Diet..

[37] McArthur L.H., Ball L., Danek A.C., Holbert D. (2018). A high prevalence of food insecurity among university students in appalachia reflects a need for educational interventions and policy advocacy. J. Nutr. Educ. Behav..

[38] Conner T.S., Brookie K.L., Carr A.C., Mainvil L.A., Vissers M.C.M. (2017). Let them eat fruit! The effect of fruit and vegetable consumption on psychological well-being in young adults: A randomized controlled trial. PLoS ONE.

[39] Parletta N., Zarnowiecki D., Cho J., Wilson A., Bogomolova S., Villani A., Itsiopoulos C., Niyonsenga T., Blunded S., Meyer B. (2017). A Mediterranean-style dietary intervention supplemented with fish oil improves diet quality and mental health in people with depression: A randomized controlled trial (HELFIMED). Nutr. Neurosci..

[40] Jacka F.N., O’Neil A., Opie R., Itsiopoulos C., Cotton S., Mohebbi M., Castle D., Dash S., Mihalopoulos C., Chatterton M.L. (2017). A randomised controlled trial of dietary improvement for adults with major depression (the ‘SMILES’ trial). BMC Med..

